# Autofluorescence Imaging and Methylene Blue‐Mediated Photodynamic Therapy for Early Detection and Management of Subclinical Actinic Cheilitis

**DOI:** 10.1002/jbio.70346

**Published:** 2026-08-31

**Authors:** Juliana Cristina Oliverio de Araújo, Og Fray Junior, Hermano Camelo Paiva, Alyne Simões Gonçalves, Martha Simões Ribeiro

**Affiliations:** ^1^ Professional Master Radiation Technology in Health Sciences Nuclear and Energy Research Institute (IPEN‐CNEN) São Paulo Brazil; ^2^ Center for Lasers and Applications, Nuclear and Energy Research Institute (IPEN‐CNEN) São Paulo Brazil; ^3^ Department of Restorative Dentistry School of Dentistry, University of São Paulo São Paulo Brazil; ^4^ Department of Biomaterials and Oral Biology School of Dentistry, University of São Paulo São Paulo Brazil

**Keywords:** blue LED, noninvasive approaches, optical techniques, red laser, solar cheilosis

## Abstract

**Objectives:**

Optical autofluorescence imaging is a noninvasive adjunct for detecting potentially malignant oral disorders, such as actinic cheilitis (AC). This study evaluated its use for early detection and for monitoring tissue response following methylene blue‐mediated photodynamic therapy (MB‐PDT).

**Methods:**

Lip autofluorescence was assessed using a handheld device (405 ± 20 nm). Areas with altered fluorescence underwent biopsy for confirmation. Patients with AC received five MB‐PDT sessions (660 nm, 100 mW, 120 s/point) after topical 1% MB. Grayscale intensity was quantified before treatment and at 30 days.

**Results:**

Of 25 screened individuals, 19 had confirmed AC, and 17 completed follow‐up. Lesions showed higher fluorescence intensity than healthy tissue. After MB‐PDT, mean intensity and variability significantly decreased, indicating tissue response. Smokers showed a smaller reduction.

**Conclusion:**

Autofluorescence imaging enables early detection and noninvasive monitoring of AC. Combined with MB‐PDT, it represents a practical strategy for managing high‐risk patients and supporting early intervention.

## Graphical abstract

Fluorescence imaging enables the detection of subclinical actinic cheilitis by revealing early optical alterations in lip tissue. Methylene blue‐mediated photodynamic therapy selectively targets dysplastic areas, enhancing treatment precision and preventing disease progression without adverse effects. This image‐guided approach improves diagnostic sensitivity and therapeutic accuracy, supporting early intervention and disease control.

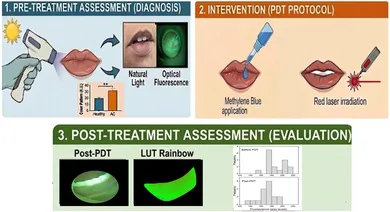

## 1 Introduction

Actinic cheilitis (AC) is a potentially malignant disorder that primarily affects the vermilion of the lower lip and results from chronic exposure to ultraviolet radiation. The lower lip is more frequently involved than the upper lip, largely due to its greater eversion and, consequently, increased solar exposure. AC is commonly associated with outdoor occupations and heightened susceptibility in individuals with genetic predispositions. Additional cofactors, such as smoking and immunosuppression, further elevate the risk of progression to squamous cell carcinoma (SCC) [1].

Clinically, AC presents with an indistinct boundary between the lip vermilion and adjacent skin, accompanied by erythema, dryness, and scaling. In advanced cases, plaques, ulcerations, and crusting may also be present [2]. However, there is currently a lack of consensus regarding diagnostic criteria, clinical procedures, biopsy indications, and the role of adjunctive tools to guide biopsy. Although 66.75% of experts consider that diagnosis can be made clinically, based primarily on inspection and palpation, histopathological confirmation remains essential for accurate diagnosis and appropriate management [3].

Despite advances in cancer therapies such as surgery and radiotherapy, oral SCC still presents high recurrence rates, ranging from 30% to 47%. Survivors often experience significant reductions in quality of life, with common sequelae including psychosocial distress, orofacial disfigurement, dysphagia, dysgeusia, hyposalivation, and tissue fibrosis [4]. These challenges underscore the urgent need for improved diagnostic strategies, earlier detection, and more effective, less invasive treatments.

Among the tools for early diagnosis, optical autofluorescence imaging has emerged as a promising noninvasive technique. Optical autofluorescence imaging shares conceptual similarities with established fluorescence‐based approaches such as quantitative light‐induced fluorescence (QLF), which has been widely applied for the detection and monitoring of early dental caries [5]. However, while QLF is a standardized method primarily validated for hard tissues, the present approach extends fluorescence‐based assessment to soft‐tissue alterations, highlighting its potential for evaluating oral potentially malignant disorders (OPMDs), such as AC.

Once AC is diagnosed, treatment is essential to prevent malignant transformation. Management options range from surgical to nonsurgical approaches, chosen based on lesion severity, anatomical considerations, and patient preferences. Vermilionectomy remains the gold standard, offering complete lesion removal and histopathological analysis, with low recurrence rates [6]. Less invasive alternatives include laser therapy (e.g., CO_2_ or Er:YAG lasers), which ablates dysplastic tissue with good cosmetic outcomes [7]. Topical treatments such as 5‐fluorouracil, imiquimod, and diclofenac gel are also used, though they require patient adherence and may cause local adverse effects [1].

Photodynamic therapy has also been used to treat AC and is considered a promising approach [8]. PDT involves the combination of a photosensitizing agent (PS), an appropriate light source, and molecular oxygen to generate reactive oxygen species that induce cell death via oxidative stress. Studies using aminolevulinic acid (ALA) or methyl aminolevulinate (MAL), precursors of protoporphyrin IX, the active PS, combined with red light or daylight, have demonstrated a complete histopathological response in approximately 50% of cases of AC. Although cosmetic outcomes are generally favorable, adverse effects such as erythema, edema, pain, and crusting have been reported [9].

Methylene blue‐mediated photodynamic therapy (MB‐PDT) is a minimally invasive treatment that has been clinically applied to a variety of oncological and non‐malignant human conditions with minimal adverse effects [10]. However, its potential for treating AC remains largely unexplored. Additionally, strategies that combine early detection with minimally invasive treatment for AC are still limited. Therefore, this study proposes an integrated light‐based approach that combines autofluorescence imaging to screen individuals with chronic occupational sun exposure and MB‐PDT for the early management of histopathologically confirmed subclinical AC. The innovative aspect of this approach lies not in autofluorescence imaging alone, but in integrating fluorescence‐guided detection with early, minimally invasive intervention before clinical disease progression.

## 2 Material and Methods

This study was conducted in two phases and involved Community Health Agents (CHAs), professionals who work in primary health care and often serve as liaisons between the community and the health system. Participants were enrolled at the Dental Specialties Center of Osasco “Brasil Sorridente*”*, Osasco, São Paulo, Brazil. The sample size was determined using the following equation for a cross‐sectional study [11]:

$$N=Z^{2}\;p\left(1-p\right)/d^{2}$$

where N is the number of individuals; p represents the expected prevalence of actinic cheilitis in populations with chronic occupational sun exposure; d is the acceptable margin of error (0.25); and Z corresponds to 1.96, the value for a 95% confidence level. Based on the high prevalence of AC reported in outdoor workers, the expected prevalence was set at 0.6 [12]. Considering a 15% dropout rate, at least 17 CHAs were required to be included in the study.

CHAs underwent an initial clinical examination after providing written informed consent, in accordance with approval from the Research Ethics Committee of the School of Dentistry, University of São Paulo (protocol no. 6211798). Anamnesis and clinical evaluation were carried out to characterize the individuals in terms of age, smoking, alcohol consumption, pre‐existing conditions, and duration of professional activity. None of the CHAs reported using lip sunscreen, and only the female participants reported using lipstick or lip gloss with some degree of sun protection.

Subjects were excluded if they were under 18 years of age, had decompensated diabetes mellitus, valvular heart disease, malignant lesions, or declined to participate in the study.

### 2.1 Phase 1

**FIGURE 1:**
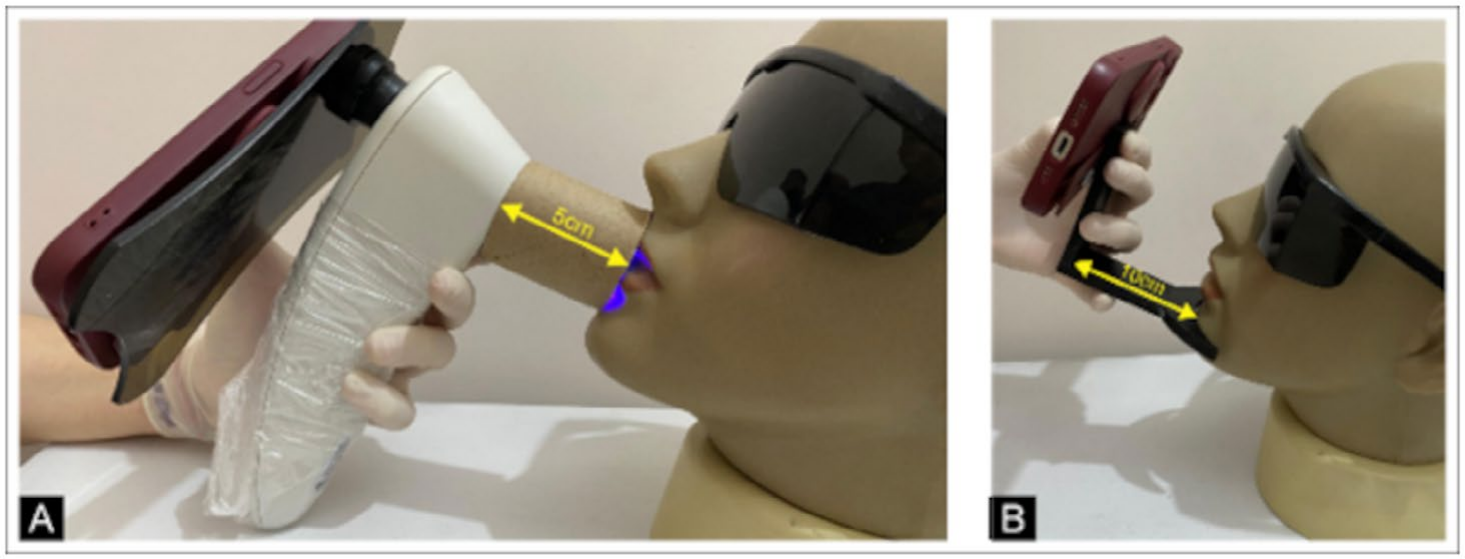
Representative images demonstrating the standardization of image capture under fluorescence (A) and natural light (B).

For optical fluorescence imaging, a violet/blue LED (light‐emitting diode) device was used with a wavelength of 405 ± 20 nm and a beam area of 0.5 cm^2^ (Evince, MMOptics, São Carlos, Brazil). The diagnosis was performed using a camera positioned above the Evince display, which includes a dedicated smartphone holder and a spacer to ensure proper focus and adequate filtering during image capture. A disposable circular device measuring 2.5 cm in radius and 5 cm in length was developed to standardize the procedure by maintaining a fixed distance between the camera and the participant's lower lip, thus ensuring image consistency (Figure 1A). The image captures were standardized at ISO 500. This value was selected to balance the sensor's sensitivity to fluorescence emission (which has lower light intensity than typical reflected light) against the need to avoid excessive digital noise, ensuring the clarity required for the identification of biofilms and lesions.

The exposure time ranged from 1/60 s to 1/40 s with exposure compensation set to 0 EV. Maintaining EV at zero, combined with the use of the EVINCE device's spacer, ensured a constant focal distance and uniform irradiance (40 mW/cm^2^) on the tissue, preventing overexposure of the fluorescent area. White balance was automatically controlled by the iPhone 13 Pro's image processing system during image acquisition, via the optical filter integrated into the EVINCE viewer. Because the device employs a narrow‐band LED excitation source (405 nm ± 20 nm) combined with a blocking filter in the viewer, the optical configuration largely restricts the detected signal to fluorescence emission from the tissue. This optical filtering acts as a hardware‐level constraint, minimizing interference from reflected excitation light and external illumination. As a result, the chromatic variations observed in the images (green for healthy tissues and red/orange for lesions) primarily reflect differences in tissue fluorescence rather than lighting artifacts.

All participants were instructed not to use any lipstick, lip gloss, or lip balm for 24 h prior to the diagnostic examination. Before image acquisition, a visual inspection was performed, and the lip surface was cleaned with dry gauze to ensure the absence of exogenous residues that could produce false fluorescence or mask the excitation light. Three photographs of each participant were taken with the iPhone 13 Pro camera from three angles: left, center, and right. The procedure was conducted in a dark room to enable complete and accurate visualization of the lips. Additionally, a photograph of the lips was captured under natural light at 10 cm (Figure 1B). Worth noting, although fine fissures may occur as normal anatomical variations, the EVINCE system detects tissue alterations in the epithelium and integument through the loss of autofluorescence. Superficial mechanical fissures do not alter the optical signature of the tissue in the same way as subclinical AC, which exhibits characteristic tissue disorganization detectable under 405‐nm excitation.

Images were standardized for size and resolution, and the regions of interest corresponding to the lip area were selected for analysis. Images were processed using ImageJ (version 1.54j) and converted from RGB to 8‐bit grayscale with the LookUp Tables > Rainbow RGB filter, enabling quantitative comparison across subjects. In this approach, grayscale values represent the processed fluorescence signal intensity, allowing comparison between healthy and altered tissues. Due to image conversion, regions showing loss of green autofluorescence appear as higher grayscale values, reflecting altered fluorescence patterns associated with tissue changes.

Following optical fluorescence imaging, individuals who exhibited alterations in green fluorescence, typically associated with healthy tissue [5], were referred for incisional biopsy. Samples were carefully collected, placed in a flask containing 10% paraformaldehyde, and sent to a specialized diagnostic service (CientificaLab, São Paulo, Brazil) for evaluation by a blinded oral and maxillofacial pathologist. Lips that maintained the expected green fluorescence pattern were considered healthy and served as controls. Participants with an AC diagnosis confirmed by histopathological analysis proceeded to phase 2 of the study.

### 2.2 Phase 2

Subjects attended five visits with 48‐h intervals between sessions, excluding weekends, over a 10‐day period. The intervention consisted of PDT mediated by MB in a 1% (m/v) solution, diluted in deionized water (Neon, São Paulo, Brazil), which was applied topically via gauze. After 10 min with the gauze placed on the lower lip, the entire mucosal surface was irradiated at three application points spaced 1 cm apart, using a diode laser (LaserDuo, MMOptics, São Carlos, Brazil), with a wavelength of 660 nm, fixed power of 100 mW, exposure time of 120 s, and energy per point of 12 J, with a beam area of 0.03 cm^2^. Participants were advised to apply lip sunscreen during the treatment and return after 30 days for follow‐up.

The data were first assessed for normality using the Shapiro–Wilk test. Subsequently, two‐sample (phase 1) and paired Student's *t*‐tests (phase 2) were performed using OriginPro 8.5 software. Differences were considered statistically significant at *p* < 0.05.

## 3 Results

**FIGURE 2:**
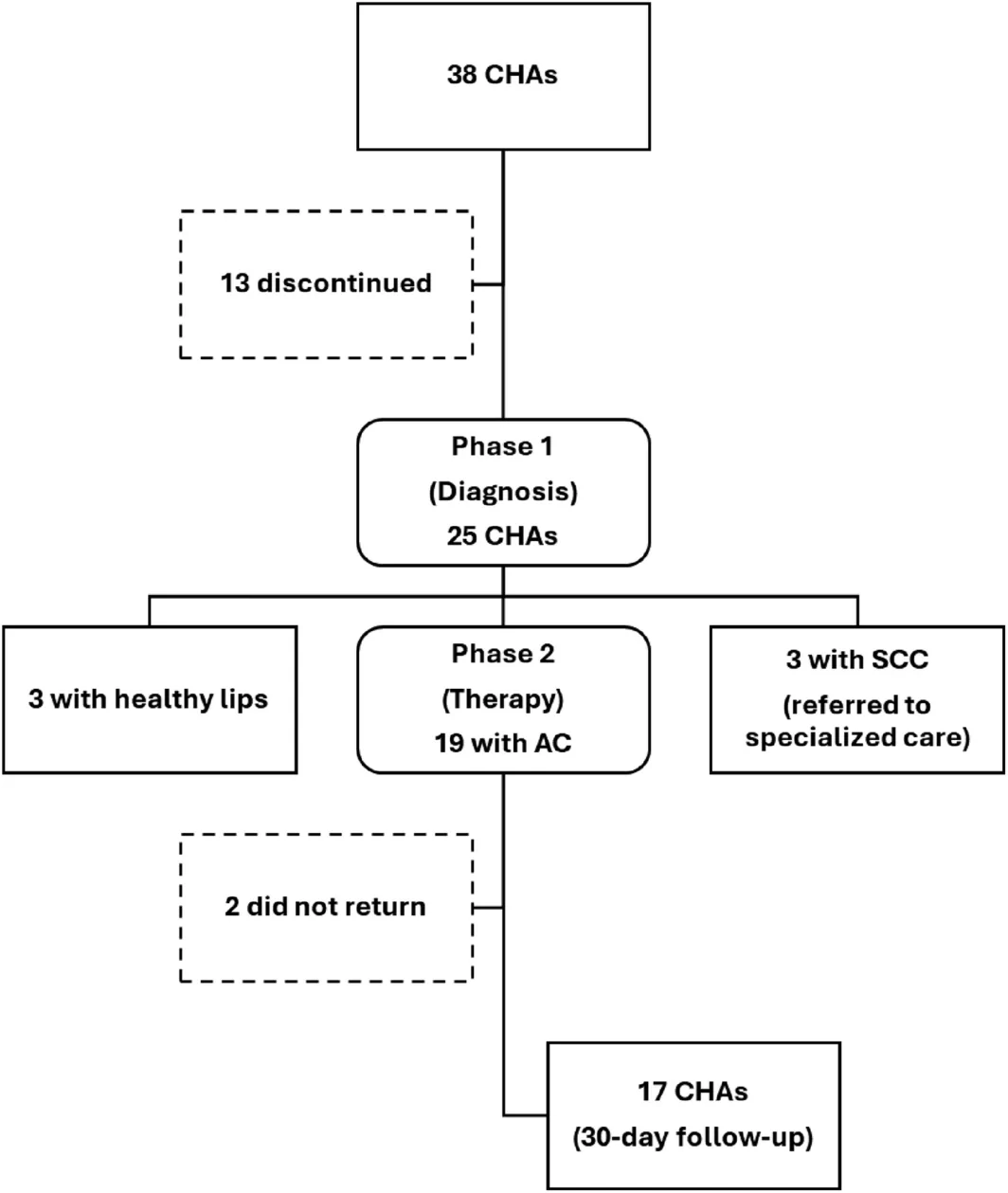
Schematic workflow illustrating the sequential steps of the experimental procedure conducted in this study. AC, actinic cheilitis; CHAs, community health agents; SCC, squamous cell carcinoma.

Thirty‐eight subjects were recruited to participate in this study. However, 13 did not attend the first visit, and 25 CHAs with a mean age of 49 years and an average of 8.6 years as a CHA were enrolled in phase 1 of the study. Clinically, no visible signs of AC were detected. Histopathological analysis revealed AC in 19 participants, AC associated with SCC in 3, and no lip lesions in the remaining 3. The individuals diagnosed with SCC were referred to the Cancer Institute of the State of São Paulo (ICESP) for specialized care, while 19 CHAs advanced to phase 2. Thirty days after treatment, 17 participants returned for follow‐up fluorescence imaging (Figure 2). No participants reported pain or burning sensations during or after the treatment sessions, and no adverse effects were observed, indicating good tolerability of the procedure. Table 1 presents the CHAs' profile, organized by study phase.

**TABLE 1:** Participant distribution and characteristics in phases 1 and 2.

Participants	Phase 1 (*n* = 25, %)	Phase 2 (*n* = 17, %)
Gender		
Female	20 (80%)	13 (76%)
Male	05 (20%)	04 (24%)
Skin color		
White	08 (32%)	08 (47%)
Brown	15 (60%)	07 (41%)
Black	02 (08%)	02 (12%)
Smoking status		
Non‐smokers	18 (72%)	12 (71%)
Smoker (cigarettes)	07 (28%)	05 (29%)

**FIGURE 3:**
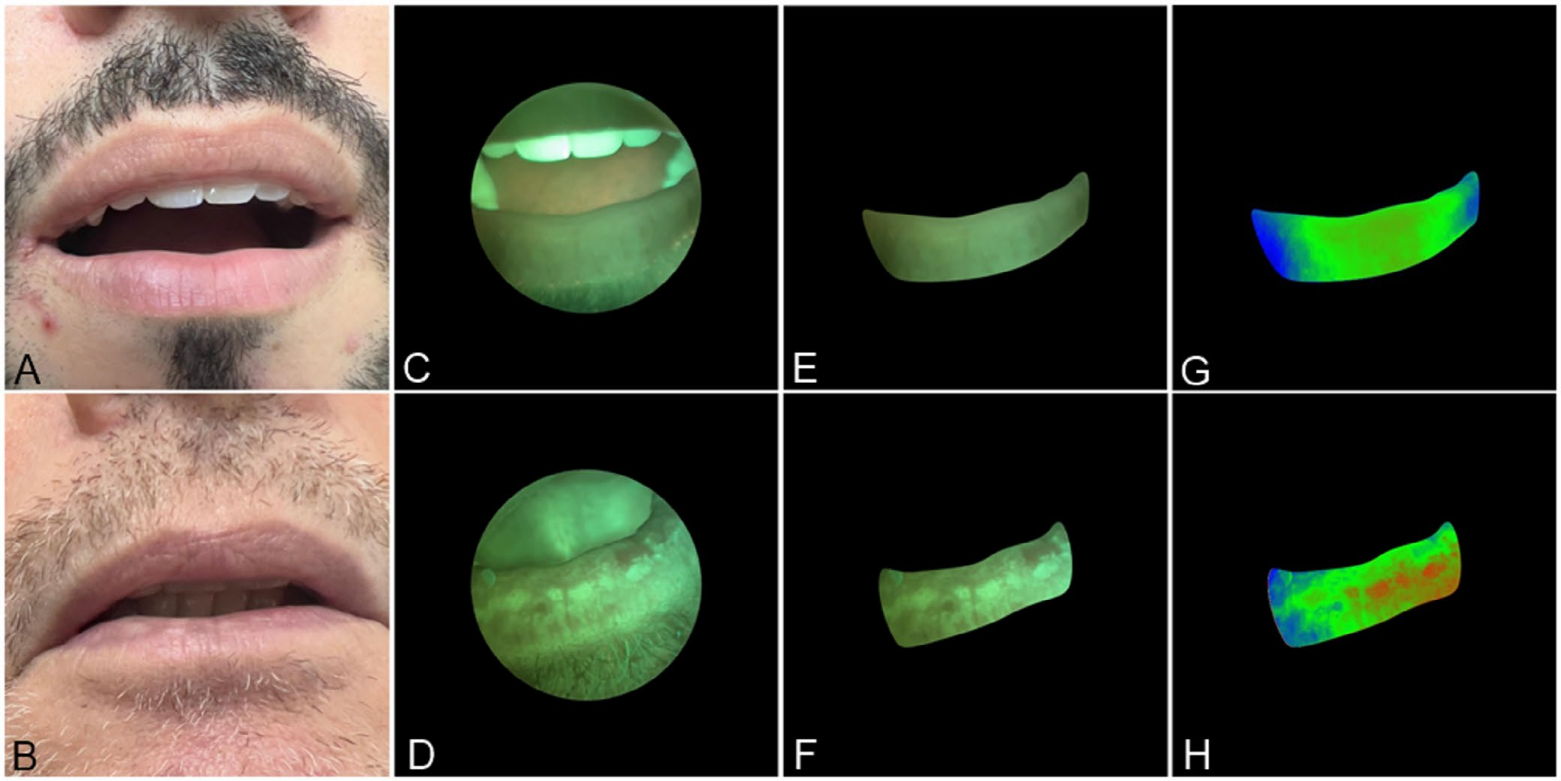
(A, B) Representative images of healthy and AC‐affected lips under natural light. (C, D) Corresponding fluorescence images of healthy and AC‐affected lips. (E, F) Cutouts from fluorescence images showing healthy and AC‐affected lips. (G, H) Same cutouts processed with the LUT Rainbow plugin in ImageJ.

Fluorescence imaging revealed that patients with AC exhibited a heterogeneous fluorescence pattern, marked by irregular, patchy areas of brighter or dimmer intensity that highlighted lesions, in contrast to the uniform autofluorescence observed in healthy lips (Figure 3).

**FIGURE 4:**
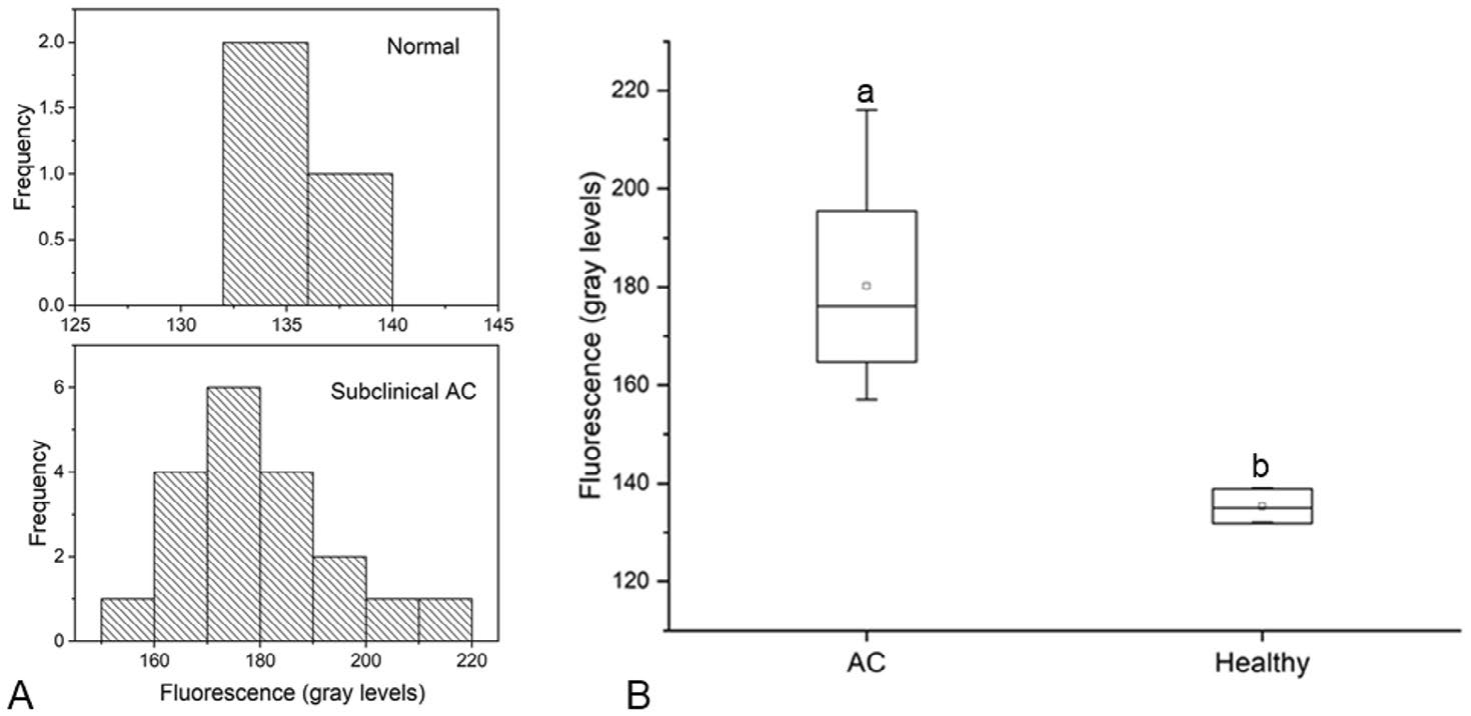
(A) Histograms representing fluorescence intensity in healthy and AC lips. (B) Box plot showing mean fluorescence (square) and median (line). The box size represents the standard deviation. Whiskers indicate the minimum and maximum fluorescence values. Different letters represent statistically significant differences (*p* < 0.05).

In healthy tissue, grayscale pixel intensities were concentrated between 132 and 140, with low‐frequency counts (0–2), indicating a narrow and homogeneous fluorescence profile. In contrast, lips with AC exhibited a broader grayscale range (150–220) and higher frequency counts (up to 6), reflecting greater variability and heterogeneity in fluorescence emission (Figure 4A). The mean grayscale intensity was 135.3 ± 3.52 for healthy lips and 180.16 ± 15.2 for lips with AC, representing a significant increase in fluorescence intensity associated with AC lesions (Figure 4B). As previously described, the increase in fluorescence intensity reflects the grayscale conversion used for image analysis, in which darker regions, associated with lower autofluorescence, are assigned higher numerical values.

**FIGURE 5:**
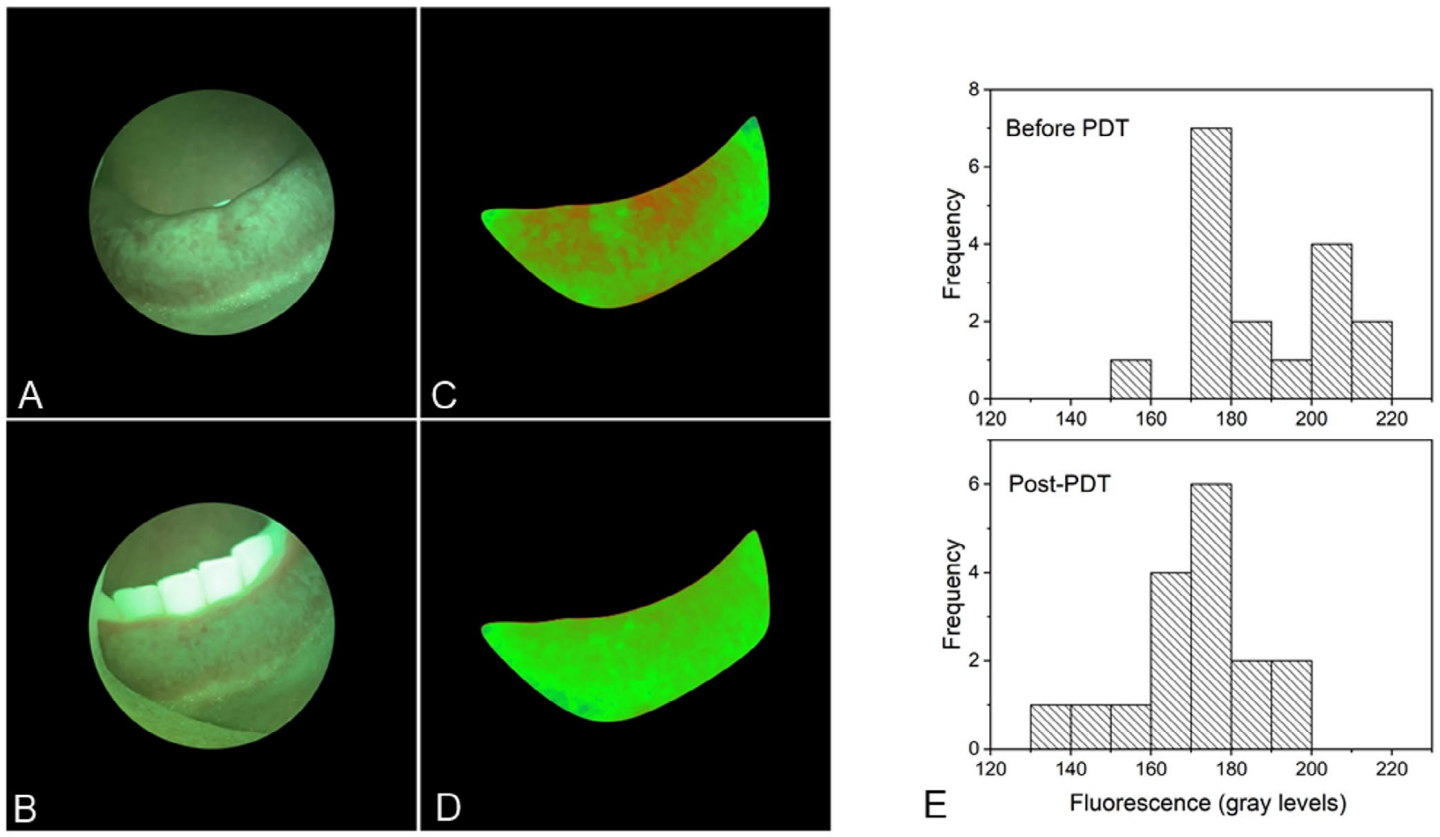
(A, B) Representative fluorescence images and (C, D) corresponding lip cutouts processed with the LUT Rainbow plugin in ImageJ software, captured pre‐ and post‐PDT, respectively. (E) Histograms representing fluorescence intensity before and after PDT.

The analysis of fluorescence intensity before and 30 days after PDT revealed a clear shift in the distribution pattern. Before PDT, fluorescence values were widely distributed, ranging from 151 to 219, with multiple peaks and several high‐intensity measurements above 200, indicating greater variability and heterogeneity in fluorescence emission. In contrast, after PDT, the values became more concentrated, ranging from 139 to 197, with a distinct peak around 170–180. The post‐treatment histogram showed lower overall intensity and a narrower distribution, suggesting a more homogeneous fluorescence profile (Figure 5).

**FIGURE 6:**
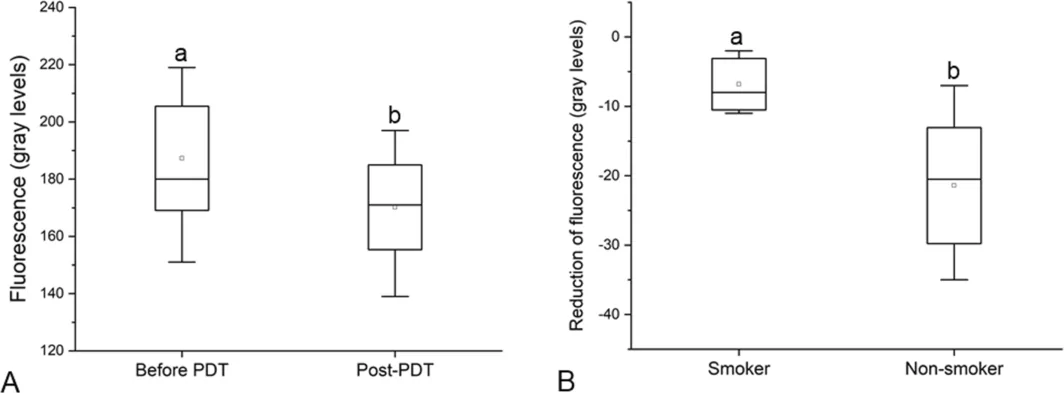
(A) Box plot displaying mean fluorescence (square) and median (line) values before and after PDT; (B) Box plot showing the reduction in mean fluorescence (square) and median (line) for smoker and non‐smoker individuals. Boxes represent standard deviation, and whiskers indicate the full range (minimum to maximum) of fluorescence values. Different letters represent statistically significant differences (*p* < 0.05).

These findings indicate a measurable decrease in fluorescence intensity and variability after PDT, suggesting optical changes consistent with tissue response, such as reduced metabolically active or abnormal tissue. Accordingly, the mean fluorescence intensity decreased significantly from 187.3 ± 18.2 before PDT to 170.2 ± 14.8 at the post‐treatment evaluation (Figure 6A). Because tobacco use is a known risk factor for AC [1], fluorescence changes were also analyzed according to smoking status among CHAs. Interestingly, smokers showed a markedly smaller mean reduction in fluorescence intensity (~4%) than non‐smokers (~11.2%) (Figure 6B). Although both groups showed a decrease in fluorescence intensity after MB‐PDT, non‐smokers exhibited a more pronounced shift toward values closer to those observed in healthy tissue. In contrast, smokers maintained higher residual fluorescence intensity after treatment, suggesting a less pronounced change in the fluorescence profile following MB‐PDT.

## 4 Discussion

This study demonstrates that optical autofluorescence imaging can identify subclinical AC in individuals with chronic sun exposure, even without clinically visible lesions, and may support monitoring tissue changes following PDT. Individuals showing altered fluorescence patterns were subsequently referred for biopsy, and AC was confirmed histopathologically in all cases. Notably, three participants were diagnosed with SCC, highlighting the potential value of autofluorescence imaging as a screening adjunct in high‐risk populations. Managing AC requires expertise in OPMDs and relies on integrating clinical examination, complementary diagnostic tools, and histopathological confirmation. In this context, dental professionals specializing in oral medicine and oral pathology play an important role in diagnosing and managing AC, particularly for lesions involving the vermilion of the lip and the oral mucosal interface. Multidisciplinary collaboration and appropriate referral pathways remain essential, especially when malignant transformation is detected.

As a non‐invasive approach, autofluorescence imaging offers several advantages, including being painless, not requiring contrast agents or tissue sampling, and being performed in real time at the point of care. Although its sensitivity and specificity for detecting OPMDs are approximately 0.8 in the specific context of AC [13], which primarily affects the lower lip, this technique can help delineate lesion margins, identify high‐risk areas for targeted biopsy, and monitor lesion progression or response to therapy [14]. Its simplicity and portability make it particularly valuable in primary care and resource‐limited settings, where early recognition is essential. While autofluorescence does not replace biopsy for definitive diagnosis, it enhances clinical evaluation, enables earlier detection of AC, and supports timely intervention to prevent malignant transformation [15]. Additionally, it serves as an effective tool for stratifying the risk of malignant transformation in oral leukoplakia [16].

Beyond autofluorescence imaging, other noninvasive optical approaches may further improve the assessment and follow‐up of AC. In vivo reflectance confocal microscopy, for example, enables real‐time visualization of tissue morphology at cellular‐level resolution and has emerged as a promising tool for evaluating epithelial alterations [17]. Although autofluorescence provides a simple, accessible method for screening and monitoring, confocal microscopy may offer complementary information on microscopic tissue changes and could be integrated into future multimodal diagnostic strategies.

Building on this diagnostic approach, we evaluated MB‐PDT as a minimally invasive treatment for lips affected by AC. We selected MB as the PS due to its established clinical use in PDT for infections, particularly in dentistry [18, 19, 20]. Beyond its antimicrobial properties via PDT, MB has been shown to downregulate matrix metalloproteinases implicated in invasion and metastasis in oral cancer and to exert therapeutic effects against various tumor types [21, 22]. Its favorable photophysical characteristics and topical application using gauze likely contributed to the absence of inflammatory responses. CHAs reported no discomfort following MB‐PDT. Moreover, the transient blue staining observed on the lips was easily removed with water.

While ALA‐ or MAL‐mediated PDT is already used as a noninvasive approach for clinically visible AC, these protocols rely on the endogenous conversion of precursors to protoporphyrin IX and may be associated with local inflammatory reactions and treatment‐related discomfort [9]. Therefore, the search continues for alternative PSs with favorable safety profiles, simplified protocols, and broader accessibility. In this context, MB‐mediated PDT is an attractive approach owing to MB's direct photosensitizing activity, wide availability, and established clinical use across various PDT applications. A previous study has reported promising in vitro efficacy of MB‐mediated PDT against multidrug‐resistant head and neck SCC and oral SCC cells [23]. Additionally, a clinical study involving 20 patients with nodular or ulcerative basal cell carcinoma reported a complete response in 11 of the 17 patients who completed treatment, with excellent cosmetic results, good tolerability, and minimal side effects [24]. These characteristics and previous findings support the potential of MB‐mediated PDT as an accessible treatment approach for community‐based and resource‐limited healthcare settings.

In our study, MB‐PDT led to detectable changes in the fluorescence pattern, including reduced mean fluorescence intensity. However, we could not confirm a complete therapeutic response because ethical constraints precluded collecting additional biopsy samples. It is also important to note that the same MB‐PDT protocol was applied uniformly across participants, without accounting for the biological variability inherent to AC. Lesions may differ in size, depth, and tissue involvement, and individual factors such as smoking status can further influence treatment outcomes.

Indeed, the five participants who were smokers showed a significantly smaller reduction in fluorescence following MB‐PDT. Because fluorescence loss is associated with tissue changes and lesion regression, this finding may reflect a diminished biological response to therapy. Tobacco use is known to impair vascularization, tissue oxygenation, and immune function [25, 26], factors that are critical for effective healing and for responses to therapies, particularly those involving photodynamic mechanisms. Furthermore, smoking can induce epithelial alterations and increased keratinization [27], leading to chronic inflammation and structural tissue changes that may modify light absorption and scattering within the tissue. These alterations may influence fluorescence intensity measurements and should therefore be considered when interpreting fluorescence‐based diagnostic or monitoring approaches in smokers. Although the smaller fluorescence reduction observed in smokers may reflect a reduced therapeutic response, smoking‐related optical or metabolic alterations could also contribute to the observed differences. These findings highlight the importance of considering smoking status when evaluating fluorescence‐based monitoring and PDT outcomes, since patient‐related factors may influence both optical measurements and biological responses to treatment, supporting the development of optimized and personalized PDT protocols.

Effective PDT requires optimized dosimetry [28], and larger or deeper lesions may demand adjustments in light dose, PS concentration, or treatment frequency to achieve adequate therapeutic responses. In the present study, treatment consisted of five MB‐PDT sessions according to a predefined treatment schedule. Although this approach was well tolerated, the number of treatment sessions may represent a clinical limitation, particularly in community‐based settings where repeated visits increase the overall treatment burden for patients and healthcare services. Uniform treatment schedules, while practical, may result in undertreatment of larger or deeper lesions or unnecessary treatment sessions for smaller or less extensive lesions, potentially affecting treatment efficacy, patient adherence, and healthcare resource utilization. Importantly, the present study was not designed to compare the therapeutic efficacy or cost‐effectiveness of MB‐PDT with established ALA/MAL‐PDT protocols. Future controlled studies should investigate whether treatment schedules adapted to individual lesion characteristics and patient profiles can achieve adequate therapeutic responses with fewer sessions while maintaining the favorable tolerability observed in this study. Such studies should also directly evaluate treatment efficacy, patient burden, and cost‐effectiveness to determine the clinical and economic value of MB‐PDT for managing AC. Although individualized approaches may require additional clinical training and implementation resources, they may support more efficient, patient‐centered PDT protocols.

This study has limitations that should be acknowledged. Although the sample size was calculated based on the expected prevalence of AC in individuals with chronic occupational sun exposure and was adequate for the proposed study design, the relatively small number of participants may limit the generalizability of the findings. Therefore, larger multicenter studies are required to validate this approach in broader populations. Additionally, although healthy individuals were included as controls during the diagnostic phase, the absence of a comparative therapeutic control group limits definitive conclusions regarding the efficacy of MB‐PDT. Furthermore, post‐treatment histopathological confirmation was not performed for ethical reasons, as additional biopsies in individuals with subclinical lesions and no clinical indication for tissue removal may not be justified. Given the invasive nature of biopsy and the generally low patient acceptance of repeated procedures performed solely for monitoring purposes, we used autofluorescence imaging as a noninvasive approach to assess tissue changes after treatment. Therefore, future controlled studies including larger cohorts, longer follow‐up periods, and histopathological assessment when clinically indicated are needed to further confirm these findings.

Despite these limitations, our findings support autofluorescence imaging as a valuable, noninvasive tool for the early detection of AC and for guiding biopsy in suspected lesions. Importantly, identifying three cases of SCC among individuals without clinically evident lesions highlights the potential value of autofluorescence imaging as a screening adjunct in high‐risk populations. Workers with chronic occupational sun exposure often remain unaware of early lip alterations, which may delay diagnosis and treatment. In this context, simple optical screening tools may facilitate earlier identification of suspicious lesions and timely referral for histopathological evaluation. Additionally, MB‐PDT was well tolerated and was associated with reductions in fluorescence intensity, suggesting tissue changes compatible with lesion response. To our knowledge, this is the first study to integrate autofluorescence‐based screening with MB‐mediated PDT to detect and early manage histopathologically confirmed subclinical AC. This light‐based approach links early diagnosis to minimally invasive intervention and may represent an accessible strategy to support preventive care for populations chronically exposed to ultraviolet radiation. Further studies are needed to optimize the MB‐PDT protocol, particularly regarding the number of treatment sessions and to establish its therapeutic efficacy and cost‐effectiveness.

## Funding

The authors have nothing to report.

## Conflicts of Interest

The authors declare no conflicts of interest.

## Data Availability

The data that support the findings of this study are available on request from the corresponding author. The data are not publicly available due to privacy or ethical restrictions.
